# Feasibility of brief psychological distress screening by a community-based telephone helpline for cancer patients and carers

**DOI:** 10.1186/1471-2407-10-14

**Published:** 2010-01-12

**Authors:** Anna L Hawkes, Karen L Hughes, Sandy D Hutchison, Suzanne K Chambers

**Affiliations:** 1 Viertel Centre for Research in Cancer Control, Cancer Council Queensland, PO Box 201, Spring Hill, Queensland, 4004, Australia; 2 School of Public Health, Queensland University of Technology, Brisbane, Queensland, Australia; 3 School of Nursing & Midwifery, The University of Queensland/Blue Care, 56 Sylvan Road, Toowong, Queensland, 4066, Australia; 4 School of Psychology, Griffith University, Brisbane, Queensland, Australia

## Abstract

**Background:**

Up to one-third of people affected by cancer experience ongoing psychological distress and would benefit from screening followed by an appropriate level of psychological intervention. This rarely occurs in routine clinical practice due to barriers such as lack of time and experience. This study investigated the feasibility of community-based telephone helpline operators screening callers affected by cancer for their level of distress using a brief screening tool (Distress Thermometer), and triaging to the appropriate level of care using a tiered model.

**Methods:**

Consecutive cancer patients and carers who contacted the helpline from September-December 2006 (n = 341) were invited to participate. Routine screening and triage was conducted by helpline operators at this time. Additional socio-demographic and psychosocial adjustment data were collected by telephone interview by research staff following the initial call.

**Results:**

The Distress Thermometer had good overall accuracy in detecting general psychosocial morbidity (Hospital Anxiety and Depression Scale cut-off score ≥ 15) for cancer patients (AUC = 0.73) and carers (AUC = 0.70). We found 73% of participants met the Distress Thermometer cut-off for distress caseness according to the Hospital Anxiety and Depression Scale (a score ≥ 4), and optimal sensitivity (83%, 77%) and specificity (51%, 48%) were obtained with cut-offs of ≥ 4 and ≥ 6 in the patient and carer groups respectively. Distress was significantly associated with the Hospital Anxiety and Depression Scale scores (total, as well as anxiety and depression subscales) and level of care in cancer patients, as well as with the Hospital Anxiety and Depression Scale anxiety subscale for carers. There was a trend for more highly distressed callers to be triaged to more intensive care, with patients with distress scores ≥ 4 more likely to receive extended or specialist care.

**Conclusions:**

Our data suggest that it was feasible for community-based cancer helpline operators to screen callers for distress using a brief screening tool, the Distress Thermometer, and to triage callers to an appropriate level of care using a tiered model. The Distress Thermometer is a rapid and non-invasive alternative to longer psychometric instruments, and may provide part of the solution in ensuring distressed patients and carers affected by cancer are identified and supported appropriately.

## Background

Cancer diagnosis and treatment is a significant life stressor resulting in psychological, social, physical and spiritual difficulties for both cancer patients and their carers [1-6]. Approximately 30% of cancer patients report ongoing clinically significant distress [5,7-9]. Although psychological intervention can reduce patient distress and improve quality of life [10], health professionals often fail to diagnose distress [9]. Barriers to health professionals detecting and referring cancer patients include a lack of time and self-confidence or experience in investigating distress and in using psychometric instruments [11]. As well, 10 to 50 percent of carers suffer from ongoing psychological morbidity [4]. Carers tend to be more focused on the patient's needs, are less likely to disclose their concerns, and only half of those with serious psychological distress seek help [2,4].

Cancer-specific telephone helplines provide a unique opportunity to screen callers for distress and they have been available throughout North America, Europe, the United Kingdom, Australia and other developed countries for over twenty years [12-14]. Cancer Council Helpline is an Australian telephone information/support service operated from the state-based Cancer Councils (non-government charitable organisations). The service is staffed by health professionals experienced in oncology, and provides cancer-specific information and practical support to those affected by cancer both over the phone and in mailed written materials [12,13]. Being tele-based, it can assist individuals who may not otherwise be reached due to geographical boundaries, poor health, or transportation issues [14,15]. In Queensland, Australia, Cancer Council Helpline services residents across the state of Queensland. On average, the service receives approximately 190 calls per week from cancer patients or carers and their most common requests include health-related information/resources, practice advice, and emotional support. During the current study, Cancer Council Helpline in Queensland incorporated the Distress Thermometer (DT) and associated Problem List (PL) [16] into routine screening protocol for cancer patients and carers. The DT is a rapid, non-invasive, acceptable, and valid alternative to longer psychometric instruments [17] and may provide part of the solution in ensuring distressed patients and carers are identified and supported appropriately. The PL identifies possible contributing factors, summarised into five categories: practical, family, emotional, spiritual, and physical. A recent meta-analysis found the DT demonstrated 77.1% sensitivity and 66.1% specificity to detect cancer-related distress, and 80.9% sensitivity and 60.2% specificity to detect depression [18]. The DT is comparable to more rigorous and comprehensive criterion measures, including the Hospital Anxiety and Depression Scale (HADS) [17,19-25]. A cut-off score of four on the DT yields optimal sensitivity and specificity in comparison with "caseness," as established by the HADS [22,23,26]. This cut-off has also identified patients reporting high levels of physical, emotional, practical, and family problems [27-29]. In previous studies, approximately 27-62% of cancer patients have met this cut-off [20,22,23,25,26,29,30]. The DT has been used in hospital and clinical contexts across a range of cancer diagnoses [16,21,22,25,29,31-34], however there are no studies that have evaluated the measure's utility in a community-based helpline setting.

Further, few studies have investigated the use of the DT with carers. In a recent study, oncology outpatients were asked to distribute a questionnaire to "the individual they designated as the family member closest to them during the course of their disease" [6]. The DT exhibited good diagnostic utility relative to the HADS [area under the curve or AUC = 0.88 relative to the HADS anxiety subscale (HADS-A) and 0.84 relative to the HADS depression subscale (HADS-D)]. A cut-off of four to five maximised sensitivity (86.2% HADS-A; 88.2% HADS-D) and specificity (71.2% HADS-A; 67.6% HADS-D). Just over 47% of those surveyed scored over the case threshold [6]. The DT has also been used to screen carers of patients with advanced malignancies, and reported high levels of distress, both before (mean = 6.96, s.d. = 3.24) and after surgery (mean = 5.83, s.d. = 2.29) [35].

An extension of the screening process is to match the level and nature of client distress to appropriate sources of care [36,37], a process that rarely occurs in routine clinical practice [36]. There is also limited information on the effect of distress screening on longer term functional or psychosocial outcomes. The Cancer Council Queensland has developed a tiered intervention model where callers are triaged to one of five levels of increasingly intensive psycho-social care based on their DT score and the operator's clinical judgement [37]. Again there is limited literature addressing the role of the DT and PL in client referral, and the few studies that have addressed referral have been largely descriptive. Studies indicate the instrument is accepted and perceived as helpful by healthcare workers. Two studies in cancer clinics found the DT and PL promoted communication between the patient and health care team, helped direct or prioritise interventions and referrals [38,39], and did not substantially burden the clinic or referral agencies [39]. Patient responses to the PL have been used for "intuitive rather than evidence based" service provision including: social services for practical or psychosocial problems; pastoral care for spiritual; nutritional, rehabilitation or symptom management for physical problems [40].

Further studies report on the validity of referrals based on DT cut-off. Prostate cancer patients with a DT cut-off of five were referred for psychiatric assessment, with 47% of participants meeting criteria for a DSM-IV disorder (i.e., adjustment disorder with mixed features of anxiety and depression, major depression in partial remission, and three had depression related to medication or their medical condition) [16]. Another study referred breast cancer patients after first recurrence with a DT greater than the cut-off score (or those with a score less than the cut-off who requested follow-up) to a three-month individually-tailored intervention, and found the intervention significantly reduced the rate of psychiatric disorders [40,41].

To our knowledge there has been no previous report on the feasibility of distress screening using valid instruments for callers to a community-based cancer helpline. Utilisation of the DT in a telephone helpline context may provide an opportunity to reach and support distressed individuals, particularly carers, not always identified in a clinical setting. Also, although the DT has been shown to be valid for distress screening amongst cancer patients, there has been limited research addressing distress screening in routine clinical practice for carers. For the first time, the current study aims to investigate the feasibility of detecting psychosocial morbidity for cancer patients and carers using the DT, followed by appropriate referral, in a community-based telephone helpline setting.

## Methods

### Participants and Recruitment

Consecutive inbound Cancer Helpline callers were invited to participate from September-December 2006. Eligible participants were: diagnosed cancer patients or carer/support people for a diagnosed cancer patient, over 18 years old, and English-speaking. Carers/support persons included immediate family members, relatives/friends that were involved in the care of a diagnosed cancer patient (hereafter referred to as 'carers'). Participants were recruited to the study by Cancer Helpline operators by providing verbal informed consent during the initial call.

### Data Collection

Operators asked all cancer patients and carers about their level of distress (using the DT [16]) during their initial call ('On a scale of zero to 10 how much distress have you been experiencing in the past week including today?'). Consenting participants in the study were then contacted by the research team as soon as possible after the initial call (within 30 days) to complete a 20 minute telephone interview to collect additional data. Additional data included: socio-demographics; cancer information; and anxiety and depression (HADS) [42]. Distress (DT) was recorded again at this time, as a control measure, to determine whether distress levels remained constant during the interval between the initial call and researcher follow-up. There were no significant differences in median distress levels measured at the two time-points for cancer patients or carers (Wilcoxon signed ranks test, p ≥ 0.20). To investigate selection bias, de-identified aggregate demographic data and baseline distress level were recorded for non-participating callers (i.e., callers who did not agree to participate in the study). There were no significant differences in age and gender between participants and non-participants. However, cancer patients were significantly more likely to participate than carers (59.9% vs 42.9%, χ^2 ^= 18.4, df = 1, p < 0.001). There was also no statistical difference between participants and non-participants in terms of proportions meeting the distress cut-off. However there was a substantial proportion of missing data for DT ratings for non-participants (33.6%).

### Measures

#### Socio-demographics and Cancer Information

Socio-demographic questions included gender, age, marital status, education level, and employment status. Participants were also asked what cancer diagnosis they (or their significant other) had received and the date of diagnosis, as well as treatments they had received for their cancer.

#### Distress

Distress was measured using the DT [16], a single item 11-point scale (0-no distress to 10-extreme distress) in a thermometer format used to rate level of distress. The associated PL asks respondents to respond to 34 items was modified to utilise over the telephone. This study asked respondents to respond only to the specified categories (practical, family, emotional, spiritual/religious, and physical problems) rather than the individual items within each category. For instance, "Have practical problems such as housing, finances, work, transport, or child care been a cause of your distress in the past week including today?" Helpline operators were trained to administer the DT and PL by qualified psychologists and they received regular in-services.

#### Anxiety and Depression

The HADS [42] contains 2 7-item subscales measuring anxiety (HADS-A) and depression (HADS-D). Each item is scored on a 4-point scale (0-3) resulting in a score ranging from 0-21 for each subscale. Subscale scores 0-7 classify participants as non-cases, 8-10 indicates borderline cases, and scores ≥ 11 indicate clinical levels. Total HADS scores (HADS-T) ≥ 15 indicate clinically significant distress. Studies have used the HADS as a criterion measure to validate the DT among cancer patients [19,20,23,25,26,37]. It has also been used to measure psychological distress among family members of cancer patients [6].

#### Tiered Model of Care

Operators triaged participants, utilising their DT score and the clinical judgement of operators, to the appropriate level of care using a tiered model of care [37]. The levels of care and corresponding DT score included: (i) Universal Care (information and advice; DT score of 0-3); (ii) Supportive Care (psycho-education, emotional support and/or triage; DT score of 0-3); (iii) Extended Care (focused counselling with psycho-education and coping skills training; DT score of 4-8); (iv) Specialist Care (narrow focus with skilled therapist; DT score of 4-8); and (v) Acute Care (broad focus, specialist services or multidisciplinary team; DT score of 9-10).

### Data Analysis

Descriptive analyses included frequencies, percentages, means and standard deviations, medians and interquartile ranges (IQR) for skewed data, to describe the characteristics of the study sample (socio-demographics and cancer information), anxiety and depression (HADS), distress (DT and PL) and triage (level of care). Receiver operating characteristic (ROC) analysis was used to evaluate the diagnostic accuracy of the DT to detect cases identified by the HADS. A series of ROC curves were created using the different DT cut-off scores on the clinical cut-off score of the HADS-T (≥ 15) and HADS-A and HADS-D (≥ 8) in order to measure the sensitivity and specificity values in discriminating cases as identified with the HADS. Differences between cancer patients and carers for demographics, cancer information, anxiety, depression and distress were analysed with t-tests, Mann-Whitney U-tests and chi-square analyses. The associations between participant characteristics and DT, DT and HADS, Problem List and level of care were assessed using chi-square analyses. Participant characteristics significantly associated with distress (p < 0.05) were entered into a multivariate logistic regression model to assess relative contributions. Quantitative data were analysed using the 'Statistical Package for Social Sciences' (SPSS) 14.0 for Windows.

### Ethics

Approval was received from the Griffith University Human Research Ethics Committee.

## Results

### Response Rate

From September to December 2006, 341/648 (52.6%) Cancer Helpline callers provided informed consent to participate. Reasons for non-participation included: 162 (25.0%) refusals; 40 (6.2%) callers were not asked to participate by operators; 53 (8.2%) callers had missing data related to their reason for refusal (indicating they refused or were not asked to participate by the operator); 49 (7.6%) did not complete the additional data collection within 30 days; and three (0.5%) callers died before the additional data collection took place.

### Participants

The majority of callers were: cancer patients (65.4%); women (76.2%); employed (41.4%) or retired (31.5%); and married or in a de-facto relationship (61.2%). Mean age was 55.9 years (SD = 12.9 years). Just over 60% (61.9%) reported senior high school, trade or technical certificate, or university as their highest level of education. Compared with cancer patients, carers were more likely to be younger (t = -4.2, df = 338, p < 0.001) and female (89.0% versus 69.5%, χ^2 ^= 16.2, df = 1, p < 0.001). Carers were also more likely to be employed casual part-time (13.7% versus 4.9%) or be involved full time in home duties/caring (22.2% versus 7.2%) and less likely to be unemployed/looking for work/permanently ill/disabled/unable to work (7.7% versus 18.4%) or retired (21.4% versus 36.8%) (χ^2 ^= 35.1, df = 5, p < 0.001).

The most commonly reported cancer types were breast (31.4%) and prostate cancer (18.2%). Cancer patients were significantly more likely to report a breast cancer diagnosis than carers (43.0% versus 21.4%, χ^2 ^= 15.7, df = 1, p < 0.001). Carers were more likely to report lung (15.4% versus 7.6%, χ^2 ^= 5.0, df = 1, p = 0.03), liver (12.0% versus 3.6%, χ^2 ^= 8.9, df = 1, p = 0.003), or brain (8.5% versus 1.8%, χ^2 ^= 8.9, df = 1, p = 0.003) cancer diagnoses. The median time since diagnosis was 2.0 months (IQR = 9.0) and there was no statistical difference between cancer patients and carers. Approximately 40% (41.8%) of callers indicated that they, or the cancer patient they cared for, had undergone surgery in the last 6 months. Further, over the last 6 months, chemotherapy, radiation and hormone therapy had commenced for 24.0%, 11.5%, and 6.3% of the total sample, respectively. Also, cancer patients were significantly more likely to report commencing chemotherapy within the last 6 months, compared to carers (27.4% versus 17.4%; χ^2 ^= 4.1, df = 1, p = 0.04).

### Psychosocial Adjustment

#### Diagnostic Accuracy of the DT

**Figure 1:**
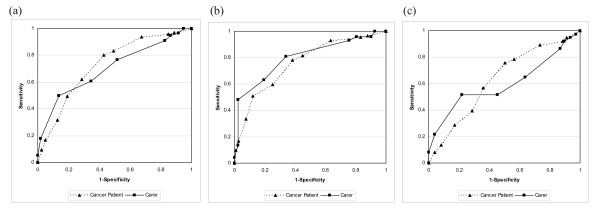
**Area under the receiver operator characteristic (ROC) curve for the criterion measures**. (a) Hospital Anxiety and Depression Scale [42] total score (≥ 15), (b) Hospital Anxiety and Depression Scale anxiety subscale (≥ 8), and (c) Hospital Anxiety and Depression Scale depression subscale (≥ 8).

ROC demonstrated the optimal cut-off score on the DT in identifying caseness according to the HADS-T cut-off (≥ 15) and HADS-A and HADS-D cut-offs (≥ 8). Sensitivity and specificity at each DT cut-off are presented in Table 1, and ROC curves are graphed in Figure 1. For cancer patients, the area under the curve (AUC) was 0.73 (95% CI 0.66-0.80) with a DT cut-off ≥ 4 maximising sensitivity and specificity in detecting general psychosocial morbidity (HADS-T score ≥ 15). This was also a reasonable cut-off for HADS-A and HADS-D. For carers, the AUC was 0.70 (95% CI 0.61-0.80), and a cut-off of ≥ 6 maximised sensitivity and specificity for the criterion measure, HADS-T. Again this was a reasonable cut-off for anxiety and depression subscales. A cut-off ≥ 4 for carers increased sensitivity to over 94% but lowered specificity to less than 14%. The DT was more accurate in predicting HADS-A than HADS-D for cancer patients and carers. For patients, the respective AUC values were 0.76 (95% CI: 0.70-0.82) and 0.63 (95% CI: 0.55-0.71) using HADS-A and HADS-D criterion measures, respectively. For carers, the AUC was 0.80 (95% CI: 0.72-0.88) and 0.60 (95% CI: 0.47-0.72), using HADS-A and HADS-D subscales as criterion measures.

**Table 1:** Sensitivity and specificity at each cut-off point on the Distress Thermometer (DT) [16,19] for the criterion measure, Hospital Anxiety and Depression Scale (HADS) [42] for cancer patients and carers.

	HADS Total Score (≥ 15)				HADS Anxiety Subscale (≥ 8)				HADS Depression Subscale (≥ 8)			
	Cancer Patient		Carer		Cancer Patient		Carer		Cancer Patient		Carer	
DT cut-off	Sensitivity	Specificity	Sensitivity	Specificity	Sensitivity	Specificity	Sensitivity	Specificity	Sensitivity	Specificity	Sensitivity	Specificity
≥ 0	100.0	0.0	100.0	0.0	100.0	0.0	100.0	0.00	100.0	0.0	100.0	0.0
≥ 1	96.8	11.1			96.5	12.2			94.6	8.8		
≥ 2	95.8	15.1	100.0	5.2	95.6	16.8	100.0	7.3	91.9	11.6	97.3	2.6
≥ 3	93.7	32.5	96.4	8.6	93.0	36.5	95.9	9.8	89.2	26.5	94.6	6.5
≥ 4	83.2	50.8	94.6	13.8	81.6	55.1	95.9	19.5	78.4	43.5	91.9	10.4
≥ 5	80.0	57.1	91.1	17.2	78.1	61.7	93.2	24.4	75.7	49.7	86.5	13.0
≥ 6	62.1	71.4	76.8	48.3	59.7	74.8	80.8	65.9	56.8	64.0	64.9	36.4
≥ 7	49.5	81.0	60.7	65.5	50.9	87.9	63.0	80.5	39.2	71.4	51.4	54.6
≥ 8	31.6	87.3	50.0	86.2	33.3	92.5	48.0	97.6	28.4	83.0	51.4	77.9
≥ 9	16.8	95.2	17.9	98.3	16.7	97.2	13.7	97.6	13.5	91.8	21.6	96.1
≥ 10	9.5	97.6	5.4	100.0	9.7	99.1	4.1	100.0	8.1	95.9	8.1	100.0
> 10	0.0	100.0	0.0	100.0	0.0	100.0	0.0	100.0	0.0	100.0	0.0	100.0

#### Distress

Median distress was 6.00 (IQR = 4), with carers reporting higher levels than patients (z = -4.2, p < 0.001). The majority of participants met the DT cut-off ≥ 4 (72.8%), although patients were more likely to fall below the cut-off compared with carers (Table 2). Participants reported emotional problems as their main cause of distress (Table 2). Carers were significantly more likely to report family and emotional concerns than cancer patients, and patients were more likely to report treatment concerns than carers. A large proportion of participants (27.6%) reported "other" concerns; that is, concerns that could not be coded into any of the five PL categories. The most common concerns identified in the 'other' option related to decision-making support or request for further information.

**Table 2:** Level of distress and associated problem list category [16,19], level of anxiety and depression[42], and level of care cancer patients and carers were triaged to by Cancer Council Helpline operators[37].

	Cancer Patient		Carer		
	*n*	*%*^1^	*n*	*%*	*p-value*
**DT cut-off^2^**					<0.001
Below cut-off (<4)	80	36.2	11	9.6	
Above cut-off (≥ 4)	141	63.8	103	90.4	
**Problem List Category**					
Practical	53	23.8	32	27.1	0.50
Family	27	12.1	32	27.1	<0.001
Emotional	115	51.6	84	71.2	<0.001
Spiritual	3	1.3	2	1.7	0.80^5^
Treatment	83	37.2	28	23.7	0.01
Symptoms	21	9.4	15	12.7	0.35
Other	61	27.4	33	28.0	0.90
**HADS: Anxiety^3^**					0.03
Normal	108	48.4	42	35.6	
Borderline	50	22.4	26	22.0	
Clinical	65	29.1	50	42.4	
**HADS: Depression^3^**					0.68
Normal	147	65.9	80	67.8	
Borderline	42	18.8	24	20.3	
Clinical	34	15.2	14	11.9	
**HADS cut-off**					0.28^5^
Below cut-off (<15)	127	57.0	60	50.8	
Above cut-off (≥ 15)	96	43.0	58	49.2	
**Level of Care triaged to^2,4^**					0.15^5^
Universal	59	27.7	21	19.1	
Supportive	111	52.1	69	62.7	
Extended	43	20.2	19	17.3	
Specialist	0	0.0	1	0.9	
Acute	0	0.0	0	0.0	

1. All percentages are valid percentages (i.e., missing values are excluded from the denominator).

2. Missing values: Distress level -1.8%; Level of Care-5.3%.

3. Anxiety and Depression subscales: Normal = score of 0-7; Borderline = score of 8-10; Clinical = score of 11+.

4. Extended, specialist and acute care categories were collapsed for chi-square comparisons.

Greater than 20% of cells have expected count less than 5 and/or minimum expected count is less than 1. Interpret with caution.

#### Participant Characteristics Associated With Distress

At the bivariate level: female gender (χ^2 ^= 5.68, df = 1, p = 0.017); younger age-group (χ^2 ^= 22.7, df = 4, p < 0.001); current employment (χ^2 ^= 14.0, df = 5, p = 0.016); higher household income (although persons with an income of $60,000 per annum to less than $80,000 indicated higher levels of distress than those on $80,000 or more; χ^2 ^= 10.4, df = 4, p = 0.034); and carer vs cancer patient (χ^2 ^= 26.8, df = 1, p < 0.001) were significantly associated with reporting a distress level of ≥ 4 at Time 1. Participant characteristics including ethnicity, health cover, country of birth, education and marital status were not significantly related to level of distress (p > 0.05). Cancer related variables including months since diagnosis and type of treatment were also unrelated to distress levels (p > 0.05). However, multivariate analysis revealed that type of caller was the only variable that maintained a statistically significant association with level of distress with carers nearly 4 times more likely to report a distress level of ≥ 4 (Table 3).

**Table 3:** Distress (score of 4 or higher on the Distress Thermometer)[16,19] by participant characteristics for Cancer Council Helpline callers.

	Proportion distressed				
Characteristic	*n*	%	Adjusted OR	95% CI	*p-value*
**Type of Caller**					0.001
Carer	103	90.4	3.90	1.78-8.57	
Cancer Patient	141	63.8	1.00	Reference	
**Gender**					0.52
Female	194	76.1	1.25	0.64-2.45	
Male	50	62.5	1.00	Reference	
**Age-group**					0.09
<40 years	37	90.2	3.08	0.70-13.54	
40-49 years	49	84.5	3.12	0.83-11.80	
50-59 years	64	66.7	1.15	0.39-3.40	
60-69 years	68	74.7	2.02	0.86-4.78	
70+ years	25	52.1	1.00	Reference	
**Work status**					0.54
Employed full-time	48	77.4	1.07	0.34-3.33	
Employed part-time	36	73.5	1.12	0.34-3.68	
Employed casual part-time	23	85.2	1.70	0.40-7.24	
Home duties or carer	33	80.5	0.96	0.30-3.06	
Unemployed^1^	40	80.0	2.42	0.85-6.85	
Retired	63	60.0	1.00	Reference	
**Household income**					0.22
<$20,000	44	62.9	0.46	0.15-1.40	
$20,000 to <$40,000	64	68.8	0.45	0.16-1.26	
$40,000 to <$60,000	41	70.7	0.46	0.16-1.30	
$60,000 to <$80,000	23	92.0	2.16	0.40-11.74	
$80,000 +	36	81.8	1.00	Reference	

1. Unemployed group includes those looking for work, permanently ill, disabled or unable to work.

#### Anxiety and Depression

Approximately 56% and 33% of participants scored above the HADS-A and HADS-D cut-off (≥ 8), respectively (see Table 2 for patient and carer breakdowns). Compared with cancer patients, carers were more likely to be anxious. However there were no differences between carers and patients in terms of depression status. Just under half (45.2%) of participants reached the HADS-T clinical cut-off ≥ 15 and there was no statistical difference between carers and cancer patients.

#### Associations with DT

A greater proportion of family, emotional, and other problems were associated with a DT cut-off ≥ 4 in cancer patients (Table 4). For carers, emotional problems were associated with increased distress. However, only a small proportion of carers fell below the distress cut-off leading to small cell sizes, hence chi-square comparisons for this group need to be interpreted with caution. Falling above the DT cut-off was strongly associated with increasing anxiety (HADS-A) for both carers and patients, and increasing depression for patients (Table 4). Again, chi-square comparisons for carers need to be interpreted with caution. Cancer patients with a DT ≥ 4 were significantly more likely to score ≥ 15 (overall cut-off) on the HADS-T. This trend was also observed for carers but the association was not significant.

**Table 4:** Associations between level of distress (Distress Thermometer, DT) and problem list categories [16,19], anxiety, depression (Hospital Anxiety and Depression Scale, HADS) [42], and level of care cancer patients and carers were triaged to (Tiered Model of Care) [37] by Cancer Council Helpline operators.

	Cancer Patient					Carer				
	DT score <4		DT score ≥ 4			DT score <4		DT score ≥ 4		
	*n*	*%*	*n*	*%*	p-value	*n*	*%*	*n*	*%*	p-value
**Problem List Category**										
Practical	15	18.8	37	26.2	0.21	3	27.3	26	25.2	0.88^3^
Family	4	5.0	23	16.3	0.01	3	27.3	28	27.2	1.00^3^
Emotional	25	31.3	89	63.1	<0.001	5	45.5	76	73.8	0.05^3^
Spiritual	1	1.3	2	1.4	0.92^3^	0	0.0	2	1.9	0.64^3^
Treatment	24	30.0	58	41.1	0.10	4	36.4	23	22.3	0.30^3^
Symptoms	5	6.3	16	11.3	0.21	2	18.2	13	12.6	0.60^3^
Other	12	15.0	48	34.0	0.002	2	18.2	30	29.1	0.44^3^
**HADS: Anxiety^1,2^**					<0.001					0.008^3^
Normal	59	73.8	48	34.0		8	72.7	33	32.0	
Borderline	13	16.3	37	26.2						
Clinical	8	10.0	56	39.7		3	27.3	70	68.0	
**HADS: Depression^1,2^**					0.005					0.70^3^
Normal	64	80.0	83	58.9		8	72.7	69	67.0	
Borderline	8	10.0	33	23.4						
Clinical	8	10.0	25	17.7		3	27.3	34	33.0	
**HADS cut-off**					<0.001					0.13
< 15	64	80.0	62	44.0		8	72.7	50	48.5	
≥ 15	16	20.0	79	56.0		3	27.3	53	51.5	
**Level of Care Triaged to**					<0.001					0.25^3^
Universal	35	46.7	23	16.9		3	30.0	18	18.8	
Supportive	36	48.0	74	54.4		7	70.0	58	60.4	
Extended-Acute	4	5.3	39	28.7		0	0.0	20	20.8	

1. Anxiety and Depression subscales: Normal = score of 0-7; Borderline = score of 8-10; Clinical = score of 11+.

2. Borderline and clinical categories have been collapsed for chi-square comparisons.

Greater than 20% of cells have expected counts less than 5 and/or the minimum expected count is less than 1. Interpret with caution.

### Psychosocial Intervention

#### Tiered Model of Care

Table 2 includes the level of care callers were triaged to when they called Cancer Helpline. The majority of callers (80.5%) received Universal Care or Supportive Care. Almost 20% were triaged to Extended Care. Only one participant was directed to Specialist Care, and no callers required Acute Care. Collapsing Extended, Specialist and Acute care, there were no significant differences between cancer patients and carers in terms of level of care received.

#### Association between Distress and Level of Care

Table 4 outlines the association between level of distress and level of care. Patients above DT cut-off ≥ 4 were more likely to receive extended or specialist care. The association between distress and level of care was not significant in the carer sample although there was a trend for highly distressed callers to be triaged to more intensive care. The chi-square associations in the carer sample were compromised by small cell sizes and must be interpreted with caution.

## Discussion

Study results indicate that a high proportion of Cancer Helpline callers (64% of cancer patients and 90% of carers) were distressed (DT ≥ 4); and Cancer Helpline presents a unique and feasible opportunity to screen cancer patients and carers for distress using the DT and to triage callers to an appropriate level of care.

The proportion of distressed cancer patients (64%) was consistent with the literature, albeit at the higher end of reported rates [23,26,30]. However, the proportion of distressed carers (90%) was considerably greater than previously reported (47%) [6], which may be explained by the study context. Previous investigators have recruited participants via oncology outpatient centres [6], whilst one might expect Cancer Helpline callers to be more distressed given they contacted the service for assistance. In addition, consistent with earlier findings [43,44], investigation of the association between participant characteristics and distress revealed that carers suffered from higher levels of distress than cancer patients. In particular, we found that carers were significantly more likely to identify emotional and family problems compared with the patient group, which may have contributed to their higher levels of distress.

ROC analyses revealed that a DT cut-off score ≥ 4 had optimal sensitivity (83%) and specificity 51%) for cancer patients relative to the HADS-T cut-off score, which has been reported previously [17,22]. In comparison, a DT cut-off ≥ 6 provided optimal sensitivity (77%) and specificity (48%) for carers. A cut-off ≥ 4 for carers, increased sensitivity to over 94%, but reduced specificity to less than 14%. Although maintaining a cut-off of ≥ 4 for carers would increase the number of false positives, it would also: ensure consistency across patient and carer groups; create less confusion for Cancer Helpline staff; and increase the sensitivity to detect distressed carers, a group that is less likely to present with psychological symptoms [2,4].

The DT displayed good overall accuracy with ROC curve analyses yielding AUC estimates relative to the HADS-T cut-off score (≥ 0.70). This is consistent with earlier findings [16,17,22,33]. However, AUC estimates for the HADS subscales indicated poor DT score discriminatory power relative to the HADS-D. Further, a DT cut-off ≤ 4 did not correlate with HADS-D in the carer group. Some studies have indicated moderate to high correlations between DT score and both HADS subscales [6,23], however there is evidence to suggest that the DT may not adequately detect depression [17,34]. Although the HADS is a well-validated measure of depression and anxiety, some researchers have suggested that distress is a more complicated concept. Trask et al. (2002) found that the HADS-T accounted for less than 20% of the variability of "distress" as measured by the DT [34]. They also reported that HADS-D scores did not significantly correlate with DT ratings. Another study, utilising the Patient Health Questionnaire 9-Item Depression Module as the criterion measure found that a DT cut-off ≥ 7 was required to detect depression [21].

DT scores were associated (although not significant for carers) with level of care referrals and HADS-T scores indicating that the DT was useful for triaging to the appropriate level of care. Requesting a DT rating was standard protocol for Cancer Helpline operators during the study. However, 30% of callers who did not participate in the study were not asked to provide a distress rating by the helpline operator. This may indicate that the operators were having difficulty utilising the DT, or the DT was considered inappropriate in particular situations. As such, ongoing training and support for Cancer Helpline operators is important in the implementation of distress screening instruments.

Study limitations included: (i) a 53% response rate, with carers less likely to respond than cancer patients, which may influence the generalisability of these results particularly in a carer population; (ii) self-reported data which limited our ability to collect clinical information (eg. cancer stage) and make comparisons with study data; (iii) the high proportion of carers reaching the DT cut-off ≥ 4, relative to those below the cut-off which made statistical comparisons unstable; (iv) HADS data was collected up to 30 days after the DT score was collected which may have impacted on the correlation between the two instruments. However statistical testing indicated no difference in median distress levels measured at the two time-points; and (v) the use of HADS as the only criterion measure, as there is some controversy over the appropriateness of this measure in assessing psychopathology in palliative populations and women with early breast cancer [20].

## Conclusion

For patients and carers affected by cancer, distress is prevalent but often under-recognized and under-treated. The results of this study suggest that a brief screening instrument, the DT, can be incorporated into a community-based telephone helpline to prospectively and rapidly identify cancer patients and carers with elevated levels of distress. However, further research is required to better understand the ability of the DT in identifying depression. Brief distress screening by a community-based cancer helpline may help to bridge the treatment gap and ensure that people who are distressed by a diagnosis of cancer are identified and receive the appropriate level of supportive care.

## Abbreviations

DT: Distress Thermometer; PL: Problem List; HADS: Hospital Anxiety and Depression Scale; HADS-T: Hospital Anxiety and Depression Scale Total Score; HADS-D: Hospital Anxiety and Depression Scale Depression Subscale; HADS-A: Hospital Anxiety and Depression Scale Anxiety Subscale; IQR: Inter-Quartile Range; AUC: Area Under the Curve; ROC: Receiver Operating Characteristic.

## Competing interests

The authors declare they have no competing interests.

## Authors' contributions

ALH, SKC and SDH developed the study concept and protocol. ALH was responsible for the implementation of the study. ALH and KLH drafted the manuscript and all authors read and approved the final manuscript.

## Pre-publication history

The pre-publication history for this paper can be accessed here:

http://www.biomedcentral.com/1471-2407/10/14/prepub
